# Clinical and Laboratory Findings That Differentiate Herpes Simplex Virus Central Nervous System Disease from Enteroviral Meningitis

**DOI:** 10.1155/2016/3463909

**Published:** 2016-08-01

**Authors:** Layli Sanaee, Monica Taljaard, Tim Karnauchow, Jeffrey J. Perry

**Affiliations:** ^1^Department of Emergency Medicine, University of Ottawa, Ottawa, ON, Canada K1Y 4E9; ^2^Clinical Epidemiology Program, Ottawa Hospital Research Institute, Ottawa, ON, Canada K1Y 4E9; ^3^Department of Epidemiology and Community Medicine, University of Ottawa, Ottawa, ON, Canada K1H 8L1; ^4^Regional Virology Laboratory, Children's Hospital of Eastern Ontario, Department of Pathology and Laboratory Medicine, University of Ottawa, Ottawa, ON, Canada K1H 8M5

## Abstract

*Background*. It can be difficult for clinicians to distinguish between the relatively benign enteroviral (EnV) meningitis and potentially lethal herpes simplex virus (HSV) central nervous system (CNS) disease. Very limited evidence currently exists to guide them.* Objective*. This study sought to identify clinical features and cerebrospinal fluid (CSF) findings associated with HSV CNS disease.* Methods*. Given that PCR testing often is not immediately available, this chart review study sought to identify clinical and cerebrospinal fluid (CSF) findings associated with HSV meningitis over a 6-year period. In cases where PCR was not performed, HSV and EnV were assigned based on clinical criteria.* Results*. We enrolled 166 consecutive patients: 40 HSV and 126 EnV patients. HSV patients had a mean 40.4 versus 31.3 years for EnV, *p* = 0.005, seizures 21.1% versus 1.6% for EnV, *p* < 0.001, altered mental status 46.2% versus 3.2% for EnV, *p* < 0.001, or neurological deficits 44.7% versus 3.9% for EnV, *p* < 0.001. CSF neutrophils were lower in HSV (median 3.0% versus 9.5%, *p* = 0.0002); median lymphocytes (87.0% versus 67.0%, *p* = 0.0004) and protein (0.9 g/L versus 0.6 g/L, *p* = 0.0005) were elevated.* Conclusion*. Our study found that HSV patients were older and more likely to have seizure, altered mental status, or neurological deficits than patients with benign EnV meningitis. HSV cases had lower CSF neutrophils, higher lymphocytes, and higher protein levels.

## 1. Introduction

Most viral CNS infections have a benign and self-limited course; however, herpes simplex virus (HSV) can cause both meningitis and potentially life-threatening encephalitis. Enteroviruses and HSV are the leading causes of viral meningitis, with the former being much more common [1–3]. HSV Type 1 (HSV-1) accounts for a substantially greater number of encephalitis cases compared to HSV Type 2 (HSV-2) [4]. Although meningitis and encephalitis are different clinical entities, they often have overlapping signs and symptoms, particularly in the case of meningoencephalitis and early in the disease course [5, 6]. Prompt intravenous acyclovir therapy in HSV encephalitis is associated with a reduction in mortality from approximately 70% to less than 20% and a substantial reduction in morbidity [7–10]. Given that it can be difficult to differentiate meningoencephalitis from meningitis at the bedside, especially in children, and that isolated HSV meningitis may evolve to meningoencephalitis, early recognition of HSV meningitis is a clinical priority to inform early treatment [5, 6, 11]. Polymerase chain reaction (PCR) of cerebrospinal fluid (CSF) is the gold-standard method for detecting viral meningitis [12–15]. However, PCR results are often not available in the emergency department or in many hospitals, which can lead to significant delays in treatment or unnecessary treatment [12]. At many hospitals, including our own, the PCR results become available to the clinician approximately 48 to 72 hours after they are obtained.

Few studies have assessed clinical and laboratory findings to help differentiate HSV from enteroviral or other viral meningitis. One small study which included 8 cases of HSV meningitis and 22 enterovirus cases found elevated CSF white blood cell counts (WBC) and elevated protein levels in HSV compared to enteroviral meningitis [16]. Another attempted to create a cost saving screening tool; however, few clinical characteristics were included and they had 33 HSV cases with no EnV comparators [17].

Our objective was to identify clinical features on history or physical examination or CSF analysis associated with HSV meningitis/meningoencephalitis. Patients with these features will be considered to be at high risk for HSV meningitis or meningoencephalitis.

## 2. Materials and Methods

### 2.1. Study Design and Setting

This chart review study assessed patients from January 2005 to December 2011, from three university-affiliated tertiary care hospitals in Ottawa, Ontario, Canada. Two were primarily adult hospitals and the third was a children's hospital. Each emergency department sees approximately 70,000 patients annually. The research ethics boards at all sites approved the study.

### 2.2. Selection of Participants

We enrolled consecutive patients 3 months of age or older with positive HSV or enteroviral meningitis confirmed by virology results or a final hospital discharge diagnosis of viral meningitis. All patients with a discharge diagnosis containing a diagnostic code for viral meningitis, meningitis, or encephalitis at one of the hospitals were screened for possible inclusion. A log containing only PCR positive results was obtained from our regional virology laboratory and also screened for inclusion. Both admitted patients and those discharged from the emergency department were included. Patients were excluded if they were less than 3 months old or had confirmed or suspected bacterial meningitis (e.g., positive culture or full course of parenteral antibiotics) or viral meningitis demonstrated by PCR due to a virus other than HSV or EnV. We excluded patients less than 3 months of age because of the age-dependent variability in CSF pleocytosis in response to viral infection [18–21].

The sample size was determined by feasibility. We sought to have a sample of greater than 100 cases of viral meningitis. Based on a quick electronic search of viral meningitis and viral encephalitis cases, it was determined that a 6-year consecutive period would provide us with enough cases, assuming that about one in six of possible cases would be included. The years assessed were the most recent with complete medical records at the time of the study. All consecutive patients during this time period were assessed for eligibility.

### 2.3. Outcome Assessment

Virology cases of enteroviral or HSV meningitis/meningoencephalitis were identified by CSF PCR analysis at our regional virology laboratory reporting (Appendix  1 in Supplementary Material available online at http://dx.doi.org/10.1155/2016/3463909) [22, 23]. Our regional virology laboratory does not differentiate between HSV-1 and HSV-2 on the final report. This has implications for clinical course, as HSV-1 is more likely to cause encephalitis and HSV-2 frequently leads to recurrence [4, 24]. Hospital discharge criteria included patients with a final diagnosis of viral meningitis or encephalitis, without PCR results, but with CSF findings consistent with viral meningitis; negative Gram stain and negative bacterial culture; no antibiotics or discontinuation of antibiotics; and clinical documentation of strong suspicion of viral meningitis. CSF findings consistent with viral meningitis were defined as CSF white blood cell (WBC) >5 × 10^6^ cells/L [25]. We did not consider the differential of the CSF WBC count given that early viral meningitis may show neutrophil predominance [26]. Clinical cases without PCR confirmation were categorized as EnV unless they had one or more of the following* a priori* findings for HSV: magnetic resonance imaging report of encephalitis, acyclovir being continued throughout admission, final discharge summary stating high likelihood of HSV, subsequent infectious disease follow-up clinic notes stating high likelihood of HSV, and any recurrent episodes of proven HSV meningitis.

### 2.4. Data Collection

A single reviewer (LS) collected data for all cases. Data were obtained from electronic medical records and paper charts and included emergency department physician assessments, nursing assessments, discharge summaries, consultant reports, and laboratory results.

Data were extracted for 30 clinical or investigational results (Table 1). Only clinical variables known to be reliably recorded (e.g., age, sex, vital signs, neuroimaging, or laboratory findings) were selected* a priori* to be collected. If a clinical variable was not explicitly classified in the documentation, the variable was left as missing data. The only exceptions to this were seizure, rash, neurological deficit, and headache. These features were believed to be well documented for patients in whom meningitis is diagnosed; therefore, if not recorded, these variables were coded as not present. For immune status, we defined* a priori* an immunocompromised state as patients with human immunodeficiency virus (HIV), immunosuppressant therapy, an organ transplant, or pregnancy. Altered mental status on history referred to changes in cognition, behavior, and/or consciousness. Neurological deficit on exam included level of consciousness, confusion, motor, sensory, or speech alterations. Nuchal rigidity included documentation of pain or stiffness on active or passive neck flexion or meningismus.

### 2.5. Statistical Analysis

Patient baseline demographics, clinical characteristics, and CSF findings were described using mean and standard deviation for continuous variables with a normal distribution, median and interquartile range for variables with a skewed distribution, and frequency or proportion for categorical variables. The distributions for continuous variables were assessed using visual inspection of histograms and normal probability plots. Differences between patients with HSV and EnV were assessed using two-sample *t*-tests or Wilcoxon two-sample tests for continuous variables and chi-squared tests or Fisher's exact tests for categorical variables. A *p* value of <0.05 was considered significant for the described tests. Planned subgroup analyses were conducted to assess if the results were consistent for PCR confirmed cases and immunocompetent cases. All data management and statistical analysis were conducted using SAS Software Version 9.2. (SAS Institute Inc., Cary, NC, USA).

## 3. Results

**Figure 1:**
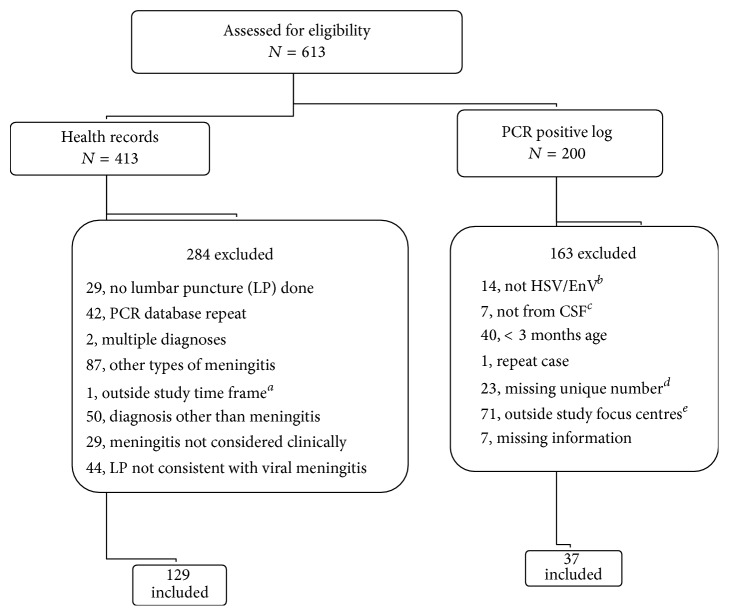
Patient flow diagram. PCR: polymerase chain reaction, HSV: herpes simplex virus, EnV: enterovirus,* a*: case prior to enrollment period of January 2005 to December 2011;* b*: further breakdown includes two human herpes virus 6, nine varicella zoster virus, one Epstein-Barr virus, one cytomegalovirus, and one toxoplasmosis;* c*: further breakdown includes four eye fluids, one nasopharyngeal, one brain biopsy, and one lymph node biopsy;* d*: hospital identification number missing from regional laboratory record, unable to cross-reference to patient's chart; e: patients from regional hospitals other than those included in the study.

During our six-year study (January 2005–December 2011) we identified 613 potentially eligible patients of which 166 patients met our eligibility criteria (Figure 1). These patients included 40 (24.1%) with HSV and 126 (75.9%) with EnV. Of the 40 patients with HSV meningitis, there were 4 patients who did not have historical or examination findings suggestive of HSV encephalitis (defined as altered level of consciousness, seizure, or focal neurological deficits). Two of these patients had PCR confirmed HSV.

Table 1 presents baseline characteristics for the included patients. Just over half (57.2%) were female, mean age was 33.5 years, 62.7% were admitted, and 1 patient (0.6%) died due to meningitis. We included 16 (9.6%) pediatric patients (age of 3 months to 17 years) and 4 (2.4%) were under the age of 1 year.

Table 2 compares characteristics of HSV and EnV meningitis patients. HSV meningitis patients were significantly older (40.4 versus 31.3 years, *p* = 0.005) and more likely to have had seizures (21.1 versus 1.6%, *p* ≤ 0.001), history of altered mental status (46.2% versus 3.2%, *p* ≤ 0.001), or neurological deficits on examination (44.7 versus 3.9%, *p* ≤ 0.001). Initial CSF findings demonstrated no significant difference in WBC counts (*p* = 0.448); however, neutrophil percentages were significantly lower in HSV cases (3.0 versus 9.5%, *p* = 0.0002), while lymphocytes (87.0 versus 67.0%, *p* = 0.0004) and protein levels (0.9 versus 0.6 g/L, *p* = 0.0005) were significantly higher. Not surprisingly, when CT, MRI, and EEG were performed, HSV patients more frequently had abnormal findings (CT head: *p* = 0.001, MRI brain: *p* ≤ 0.001, and EEG: *p* ≤ 0.001). Sensitivity analysis after removing the 16 pediatric patients did not result in significantly different results (Appendix  2 in Supplementary Material). Analysis of just the 16 pediatric patients with only one HSV patient was not conducted due to the small numbers.

Our planned subgroup analysis of only virology PCR confirmed cases (HSV, *N* = 29 versus EnV, *N* = 19) had findings consistent with all patients in our study. Statistically significant associations were detected, indicating higher age, more females, higher prevalence of altered mental status and neurological deficits, lower percentages of neutrophils, and higher lymphocyte percentage and protein levels among HSV patients (Table 3). Likewise, our second subgroup analysis using only immunocompetent cases with PCR confirmed viral meningitis (HSV, *N* = 23 versus EnV, *N* = 19) had results consistent with the full study population (Appendix  3 in Supplementary Material). Receiver operator characteristic curves were calculated for cerebrospinal fluid protein, percentage of neutrophils, and percentage of lymphocytes (Appendix  4 in Supplementary Material).

## 4. Limitations

A single reviewer (Layli Sanaee) determined patient eligibility and collected the data. While there is a potential for error in the identification of eligible patients, our inclusion and exclusion criteria were defined* a priori* which minimized the risk of selection bias. Further, the virology laboratory was able to provide a definitive list of all CSF samples with positive PCR during the study period for patients at the study hospitals. This group of patients would be virtually free of any misclassification or selection bias. Given that our subgroup analysis of only these patients provided consistent results with all patients, we do not believe that the magnitude of any potential error in identification of patients is large or more likely for either the HSV group or the EnV group.

There is a potential for misclassification of historical or physical exam findings. We attempted to minimize this by only collecting clinical features that were deemed to be both potentially important and known to be consistently recorded. Complete information was obtained for the vast majority of patients with just 6.0% missing variables in the CSF analysis and 6.6% missing clinical data. For most variables, if we could not clearly determine that they were assessed, they were left as missing. The only exceptions to this were seizure, rash, neurological deficit, and headache. For these few clinical findings, if they were not explicitly stated in the patient record, they were deemed to be not present. These findings are reliably recorded for patients with meningitis that we do not believe that misclassification is likely. In the case of headache, it was present in 94.8%, which minimizes the impact of any potential misclassification. If there was any misclassification, we believe it to be nondifferential and would in fact lead to an underestimation in the clinical differences between HSV and EnV cases.

“Since PCR was not performed for all cases, the majority of cases were classified based on clinical criteria. It is important to note the inherent potential confounding in the full study analysis, as several clinical and imaging findings being measured were part of the case definition of HSV meningitis/meningoencephalitis. As a result, it is possible that some of the statistically significant differences we observed may be spurious or exaggerated. However, subgroup analysis of only the PCR confirmed cases yielded results consistent with the overall analyses. Also, the likelihood of abnormal advanced imaging such as MRI is biased toward the HSV cohort, as patients with seizure or neurological findings are more likely the ones to have received the test. However, abnormal MRI findings are an established feature of HSV encephalitis and would not be expected in EnV meningitis cases [1].”

Although there are several causes of aseptic meningitis, the most common etiology is viruses and more specifically enteroviruses [1–4]. Thus, it is reasonable to deduce that by only including cases with the clinical diagnosis of viral meningitis the majority of PCR unconfirmed cases would be due to EnV. For this reason such cases were included with the PCR EnV confirmed group in the full analysis. It is possible that viruses other than EnV with similar clinical presentations, such as Epstein-Barr Virus or adenovirus, were included in the group. Since the full analysis and PCR confirmed subgroup analysis had similar results, this was likely not a significant number of cases. Furthermore, the treatment of these relatively benign causes is also symptomatic rather than specific antiviral therapy being indicated.

Another possible limitation was that the analysis was not stratified by the timing of the lumbar puncture (LP) from the onset of illness. It was not possible to accurately collect this information in this study as it was not consistently documented. This can potentially affect results as the relative proportions of neutrophils and lymphocytes in CSF depend on the duration of illness [15, 26]. However, the timing of the LP was unlikely to be substantially different between the HSV and the EnV groups.

It is not known what proportion of the PCR confirmed HSV cases were HSV-1 versus HSV-2. Although management is the same for both, their clinical features and imaging findings can be different as HSV-2 only accounts for 1.6% to 6.5% of all herpes simplex encephalitis cases in adults [4]. Thus, the ratio of HSV-1 : HSV-2 among the cases can affect the results by influencing the proportion of meningitis and encephalitis cases in the study. It is therefore unknown in what direction the results are potentially skewed.

Finally, several other serious causes of encephalitis were not included in this study, such as Varicella Zoster Virus and West Nile Virus. The purpose of the study was to compare the most common serious causes of viral meningitis/meningoencephalitis, HSV, with the most common cause of viral meningitis, EnV. Assessing less common causes of viral meningitis, while potentially worthwhile, was beyond the scope of this study given that this study would have been grossly underpowered to find any meaningful differences between these very rare causes of serious meningitis and EnV. We chose not to combine all serious etiologies together, as we do not know if the clinical or laboratory findings would be similar for all etiologies of serious viral meningitis.

## 5. Discussion

Our six-year multicenter study identified that viral meningitis patients with HSV were more likely to be older and have seizure, an altered mental status, neurological deficits, lower CSF neutrophil counts, higher CSF lymphocyte counts, or higher CSF protein levels than patients with EnV. These findings are clinically important as physicians performing a lumbar puncture to rule out central nervous system infection, who diagnose their patients with viral meningitis, need to consider HSV meningitis. Patients with one or more of our clinical or CSF findings ought to be started on intravenous antiviral agents (e.g., acyclovir) pending the results of PCR testing.

Prior studies, including the study by Ihekwaba, have demonstrated that HSV meningitis cases have greater CSF white blood cell counts and protein levels [13, 14]. Our study did not find a statistically significant difference in the overall white blood cell count; however, we did find significant differences in the neutrophil and lymphocyte differentials. This may be due to a difference in timing of the LP in the course of illness between groups or study populations, as children tend to have LPs earlier during admission, and Ihekwaba et al. excluded patients <16 years of age [15, 27]. It may also be that the previous study sample sizes were too small and the statistical difference the researchers observed was due to chance. Similar to these two studies, we found that HSV meningitis cases had a higher protein level than EnV cases. Our study covered three emergency departments and allowed for analysis of a larger number of HSV and EnV cases than previous studies.

Another previous study, by Hanson and colleagues, developed a laboratory-screening tool for HSV meningitis using 1,659 HSV PCR requests [17]. Their tool incorporated CSF parameters along with immune status and age. No comparator group was used and they did not assess the association of clinical features with HSV in patients thought to have viral meningitis. In their study, 7.8% of patients who were not tested by their criteria were treated with intravenous acyclovir due to concern for HSV. Our study's objective was to determine high-risk features of HSV versus the more common EnV meningitis.

The majority of signs and symptoms did not differ amongst the groups, including vital signs, presence of nuchal rigidity, rash, or photophobia. We did, however, find a significant difference in the frequency of seizure, history of altered mental status, and neurological deficit on physical exam. These three features are well-known signs of encephalitis, and given that HSV has a greater propensity for progressing to encephalitis than EnV, these were not surprising results.

In the subgroup analysis of PCR confirmed cases, the HSV group had a significantly increased percentage of females. This may be accounted by the fact that HSV-2 meningitis seems to affect a greater proportion of females compared to males [24, 28, 29]. This trend was not seen perhaps as the full study analysis included a greater proportion of HSV-1 cases.

## 6. Clinical Implications

The clinical features that were statistically different between HSV and enteroviral meningitis included typical signs and symptoms of encephalitis. However, these did not identify all HSV cases. This suggests that in the absence of clinical findings we cannot absolutely rule out HSV meningitis. This has been previously found in children where HSV meningitis and meningoencephalitis can present without any overt clinical signs of meningeal irritation [5, 11]. The patients with HSV CNS disease not identified in our study by clinical or historical features ranged from 27 to 50 years of age. CSF results in addition to clinical features can help us identify high-risk patients earlier. Given that current practice in many institutions involves a significant delay to PCR testing of CSF, our study supports empiric intravenous antiviral therapy patients with suspected HSV encephalitis, including those with any of the following features: altered mental status, seizure, neurological deficits, lower CSF neutrophil percentages, high CSF lymphocyte percentages, or high CSF protein levels. Furthermore, our study supports future prospective observational studies to confirm the reproducibility of these associations and also to derive cutoffs for CSF lymphocyte proportions and protein levels. Since no absolute cutoffs for CSF parameters were derived, the diagnosis of HSV CNS disease remains clinical until PCR virology is obtained.

## 7. Conclusion

In summary, we found that patients with HSV meningitis were more likely than patients with enteroviral meningitis to present with seizure, altered mental status, neurological deficits, lower CSF neutrophils, higher CSF lymphocytes, or higher CSF protein levels. We recommend that HSV directed intravenous antiviral treatment be strongly considered in patients with one or more of these clinical or CSF features while awaiting PCR results.

## Supplementary Material

Supplementary Materials for laboratory procedures for polymerase chain reaction testing(Appendix 1); comparison of demographic, clinical characteristics and investigations between patients with herpes simplex virus and enterovirus, excluding pediatric patients (Appendix 2); univariate sensitivity analysis of cerebrospinal fluid of confirmed herpes simplex virus patients to confirmed enterovirus patients in immunocompetent patients (Appendix 3); and receiver operating characteristic curves for cerebrospinal fluid neutrophils, lymphocytes and protein in HSV meningitis/meningoencephalitis (Appendix 4).

## Figures and Tables

**Table 1:** Characteristics of all included viral meningitis patients (*N* = 166).

	Total (%)
*Demographics*	
Age (mean, SD)	33.5 (15.6)
Female	95 (57.2)
Admitted	104 (62.7)
Returned to emergency department <2 weeks	27 (16.3)
Immunocompromised	15 (9.0)
Death attributed to meningitis	1 (0.6)
*Virology *	
Confirmed HSV	29 (17.5)
Suspected HSV	11 (6.6)
Confirmed EnV	19 (11.4)
Suspected EnV	107 (64.5)
*History*	
Headache	157 (94.8)
Nausea/vomiting (*N* = 153)^*∗*^	115 (69.3)
Altered mental status	22 (13.3)
Neck pain (*N* = 144)^*∗*^	106 (63.9)
Photophobia (*N* = 130)^*∗*^	81 (48.8)
Seizure	10 (6.0)
*Physical exam*	
Neurological deficits	22 (13.3)
Rash	24 (14.5)
Nuchal rigidity, pain with flexion (*N* = 152)^*∗*^	77 (46.4)
Mean systolic blood pressure (SD)	127.9 (16.6)
Mean diastolic blood pressure (SD)	73.2 (11.8)
Mean heart rate (SD)	90.2 (19.2)
Mean respiratory rate (SD)	18.3 (4.7)
Mean temperature (SD)	37.4 (1.1)
*Investigations performed*	
Computed tomography head	115 (69.3)
Magnetic resonance imaging brain	31 (18.6)
Electroencephalogram	11 (6.6)

Data presented as *n* (%) or mean ± SD: standard deviation.

*∗* identifies where frequency is <166, as information was not documented. EnV: enterovirus; HSV: herpes simplex virus.

**Table 2:** Comparison of demographic and clinical characteristics and investigations between patients with HSV and EnV (*N* = 166).

	Total (%)		*p* value
	HSV (*N* = 40)	EnV (*N* = 126)	
*Demographics*			
Age (mean, SD)	40.4 (18.3)	31.3 (14.0)	**0.005**
Female	25 (62.5)	70 (55.6)	0.439
Admitted	40 (100)	64 (50.8)	**<0.001**
Returned to emergency department <2 weeks	8 (17.9)	19 (15.1)	0.467
Immunocompromised	6 (15.0)	9 (7.1)	0.201
*History*			
Headache	35 (97.2)	122 (99.2)	0.403
Nausea/vomiting (*n* = 153 (36,117))	27 (75.0)	89 (75.2)	0.979
Altered mental status	18 (46.2)	4 (3.2)	**<0.001**
Neck pain (*n* = 144 (32,112))	25 (78.1)	81 (72.3)	0.511
Photophobia (*n* = 130 (24,106))	16 (66.7)	65 (61.3)	0.626
Seizure	8 (21.1)	2 (1.6)	**<0.001**
*Physical exam*			
Neurological deficit	17 (44.7)	5 (3.9)	**<0.001**
Rash	6 (15.0)	18 (14.6)	0.955
Nuchal rigidity or pain with flexion (*n* = 152 (33,119))	19 (57.9)	58 (49.2)	0.489
Mean systolic blood pressure (SD)	129.9 (17.0)	127.2 (16.5)	0.518
Mean diastolic blood pressure (SD)	75.5 (12.8)	72.4 (11.4)	0.225
Mean heart rate (SD)	93.3 (19.0)	89.1 (19.3)	0.175
Mean respiratory rate (SD)	18.6 (4.4)	18.2 (4.8)	0.684
Mean temperature (SD)	37.5 (1.2)	37.3 (1.1)	0.388
*Investigations *			
CT head abnormal	3 (7.5)	0 (0.0)	**0.001**
MRI brain abnormal	13 (33.3)	3 (2.4)	**<0.001**
EEG abnormal	6 (15.0)	1 (0.8)	**<0.001**
CSF analysis (median, IQR^*∗*^)			
RBC (×10^6^/L)	5.5 (1.0–16.5)	6.0 (1.0–22.0)	0.965
WBC (×10^6^/L)	199.0 (75.0–406.0)	156.5 (51.0–420.0)	0.448
% neutrophils	3.0 (0.0–8.0)	9.5 (2.0–37.5)	**0.0002**
% lymphocytes	87.0 (72.0–94.0)	67.0 (40.0–87.0)	**0.0004**
% monocytes	7.0 (4.0–15.0)	10.0 (3.0–20.0)	0.767
Glucose (mmol/L)	3.2 (2.8–4.4)	3.1 (2.7–3.5)	0.093
Protein (g/L)	0.9 (0.6–1.2)	0.6 (0.5–0.9)	**0.0005**

Data presented as *n* (%) or mean ± SD (standard deviation). *∗* identifies where frequency is <166, as information was not documented. EnV: enterovirus, HSV: herpes simplex virus.

**Table 3:** Subgroup analysis comparing demographic and clinical characteristics and investigations between patients with confirmed HSV and confirmed EnV (*N* = 48).

	Total (%)		*p* value
	HSV (*N* = 29)	EnV (*N* = 19)	
*Demographics*			
Mean age ± SD	43.9 ± 18.4	13.1 ± 9.0	**<0.001**
Female	21 (72.4)	5 (26.3)	**0.002**
Admitted	29 (100)	17 (89.5)	0.152
Returned to emergency department <2 weeks	5 (17.2)	3 (15.8)	1.000
Immunocompromised	6 (15.0)	9 (7.1)	0.201
*History*			
Headache (*n* = 41 (25,16))	24 (96.0)	16 (100)	1.000
Nausea/vomiting (*n* = 43 (26,17))	17 (65.4)	12 (70.6)	0.722
Altered mental status	12 (41.4)	1 (5.6)	**0.008**
Neck pain (*n* = 39 (23,16))	17 (73.9)	13 (81.3)	0.711
Photophobia (*n* = 30 (17,13))	10 (58.8)	6 (46.2)	0.491
Seizure	6 (21.4)	1 (5.3)	0.215
*Physical exam*			
Neurological deficit	10 (35.7)	1 (5.3)	0.032
Rash	3 (10.3)	8 (42.1)	**0.016**
Nuchal rigidity, pain with flexion (*n* = 42 (24,18))	14 (58.3)	9 (50.0)	0.591
Mean systolic blood pressure (SD)	127.7 (17.6)	116.7 (15.2)	0.031
Mean diastolic blood pressure (SD)	75.3 (14.3)	67.3 (13.7)	0.068
Mean heart rate (SD)	96.9 (20.0)	99.1 (29.0)	0.775
Mean respiratory rate (SD)	19.0 (5.1)	22.1 (10.3)	0.244
Mean temperature (SD)	37.5 (1.2)	37.8 (1.10)	0.374
*Investigations*			
CT head abnormal	3 (10.3)	0 (0)	**<0.001**
MRI brain abnormal	9 (32.1)	1 (5.3)	**0.031**
EEG abnormal	2 (6.9)	1 (5.3)	0.224
CSF analysis (median, IQR^*∗*^)			
RBC (×10^6^/L)	5.0 (1.0–13.0)	9.0 (3.0–14.0)	0.386
WBC (×10^6^/L)	207.0 (39.0–403.0)	150.0 (42.0–365.0)	0.697
% neutrophils	3.5 (0.0–0.08)	20.0 (3.0–70.0)	**0.004**
% lymphocytes	87.0 (71.0–94.0)	55.0 (30.0–73.0)	**0.001**
% monocytes	8.0 (4.0–19.0)	11.5 (7.0–20.0)	0.444
Glucose (mmol/L)	3.3 (2.8–4.9)	3.3 (2.6–3.3)	0.141
Protein (g/L)	0.7 (0.6–1)	0.4 (0.3–0.7)	**0.001**

Data presented as *n* (%) or mean ± SD (standard deviation). *∗* identifies where frequency is <166, as information was not documented. EnV: enterovirus, HSV: herpes simplex virus.
