# Supplementary material for: Clinical and Laboratory Findings That Differentiate Herpes Simplex Virus Central Nervous System Disease from Enteroviral Meningitis
Source: Can J Infect Dis Med Microbiol. 2016 Aug 1;2016:3463909. doi: 10.1155/2016/3463909 (PMC4983658; doi:10.1155/2016/3463909)

Appendix 1: Laboratory Procedures for PCR Testing

HSV PCR and EnV RT-PCR

Total nucleic acid was extracted using the Roche MagNA LC Total Nucleic Acid Isolation Kit 1 (Roche Diagnostics, Laval, QC) and the Roche MagNA Pure Compact instrument. Total nucleic acid was extracted from 200 µL of CSF, and eluted in 100 µL (for HSV) or 50 µL (for EnV) of elution buffer. Extracted nucleic acids were stored at -20°C (for HSV) and at -80°C (for EnV) until analyzed.

HSV Real-Time PCR

Real time PCR detection of HSV was performed essentially as described by Jerome *et al*.(24)

Primers and probes were duplexed for HSV and Glyceraldehyde 3- Phosphate Dehydrogenase (GAPDH). HSV-1 and -2 consensus primers and probe described by Jerome *et al.* amplify and detect a 124bp segment of the glycoprotein B (gB) gene: forward primer CCG TCA GCA CCT TCA TCG; reverse primer CGC TGG ACC TCC GTG TAG TC. Probe was labeled at the 5' end with 6-FAM and quenched at the 3'end with Black Hole Quencher-1: CCA CGA GAT CAA GGA CAG CGG CC (Integrated DNA Technologies Coralville, IA). GAPDH consensus primer and probe sequences provided by Bio-Rad (Rio-Rad Laboratories, Montreal, QC) amplify and detect a 154 bp portion of the GAPDH gene: forward primer GAA GGT GAA GGT CGG AGT; reverse primer CAT GGG TGG AAT CAT ATT GGA A. Probe was labeled at the 5' end with TexasRed and quenched at the 3' end with Black Hole Quencher-2: CAA CGG ATT TGG TCG TAT T (Integrated DNA Technologies). Working concentrations of primers and probes were 10µM and 2µM, respectively.

Duplexed amplification mixtures were prepared using the QuantiTect Multiplex RT-PCR NoROX kit (Qiagen, Mississauga, ON): 15.0µL of 2x QuantiTech Multiplex RT-PCR NoROX Master Mix, 1.0µl Uracil-N Glycosylase (Roche), 0.5µL of each primer, and 1.25µL of probe, combined with 10µL total nucleic acid. Amplification was performed using the ABI 7500 Fast Real-Time PCR system (Life

Technologies, Carlsbad, CA) with cycling parameters: 20˚C for 10 min (1 cycle); 95˚C for 15 min (1 cycle); 95˚C for 15 s followed by 61˚C for 30 s, and 72˚C for 30 s (40 cycles). Data were analyzed using SDS software v1.4 (Applied Biosystems; Life Technologies).

EnV Real Time Reverse Transcriptase (RT) - PCR

Real time RT-PCR detection of EnV was performed essentially as described by Verstrepen *et al*.(25) EnV consensus primers and probe previously describe by Verstrepen *et al*. amplify a 145bp segment of the 5' untranslated region (5'-UTR): forward primer CCC TGA ATG CGG CTA ATC C; reverse primer ATT GTC ACC ATA AGC AGC CA. Probe was labeled at the 5' end with 6-FAM and quenched at the 3'end with Black Hole Quencher-1: AAC CGA CTA CTT TGG GTG TCC GTG TTT C (Integrated DNA Technologies). Working concentrations of primer and probe were 10µM. Amplification mixtures were prepared using Ambion Ag-path ID one step RT-PCR kit (Life Technologies) as per kit specifications: 12.5µL 2x RT-PCR buffer, 1.0µl 25X RT-PCR enzyme, 1.67µL detection enhancer, 1.0µL of each primer, 0.3µL of probe, and 2.53µL of water, combined with 5µL total nucleic acid. Amplification was performed using the ABI 7500 Fast Real-Time PCR system (Life Technologies) with cycling parameters: 45˚C for 10 min (1 cycle); 95˚C for 10 min (1 cycle); 95˚C for 15 s followed by 60˚C for 45 s (45 cycles). Data were analyzed using SDS software v1.4 (Applied Biosystems).

Appendix 2: Comparison of demographic and clinical characteristics, and investigations between patients with HSV and EnV excluding pediatric patients (N = 150)

	Total (%)		
**Demographics**	**HSV (N=39)**	**EnV (N= 111)**	**p-value**
Age (Mean, SD)	41.5 (17.3)	34.2 (12.0)	**0.019**
Female	25 (62.5)	65 (58.6)	0.543
Admitted	39 (100)	50 (45.1)	**<0.001**
Return to Emergency Department <2weeks	7 (18.0)	17 (15.3)	0.700
Immunocompromised	6 (15.4)	9 (8.1)	0.219
**History**			
Headache	35 (97.2)	110 (99.1)	0.431
Nausea/Vomiting (n = 138 (35,103))	27 (77.1)	79 (76.7)	0.957
Altered mental status	19 (48.7)	2 (1.8)	**<0.001**
Neck pain (n = 132 (32,100))	25 (78.1)	70 (70.0)	0.373
Photophobia (n = 120 (24,96))	16 (66.7)	60 (62.5)	0.705
Seizure	7 (18.0)	1 (0.9)	**<0.001**
**Physical exam**			
Neurological deficit	17 (43.6)	4 (3.6)	**<0.001**
Rash	6 (15.4)	11 (10.2)	0.391
Nuchal rigidity or pain with flexion (n = 138 (34,104))	19 (55.9)	51 (49.0)	0.488
Mean systolic blood pressure (SD)	130.2 (17.1)	129.0 (16.1)	0.704
Mean diastolic blood pressure (SD)	75.3 (13.0)	73.5 (10.9)	0.388
Mean heart rate (SD)	92.4 (18.2)	87.4 (16.5)	0.128
Mean respiratory rate (SD)	18.2 (3.6)	17.5 (2.3)	0.321
Mean temperature (SD)	37.4 (1.2)	37.3 (1.1)	0.399
**Investigations**			
CT head abnormal (test done for n=113 (33,80)	2 (5.1)	0 (0.0)	**0.066**
MRI brain abnormal (test done for n=31 (20,11)	13 (34.2)	3 (2.7)	**<0.001**
EEG abnormal (test done for n=11 (10,1)	6 (15.4)	1 (0.9)	**0.001**
CSF analysis (Median, IQR*)			
RBC (x 10^6 /L)	6.0 (1.0 – 17.0)	5.0 (1.0 – 24.0)	0.765
WBC (x 10^6 /L)	207 (80.0 – 409.0)	157.0 (51.0 – 420.0)	0.349
% Neutrophils	2.5 (0.0 – 8.0)	9.0 (2.0 – 33.0)	**0.0005**
% Lymphocytes	87.5 (73.0 – 94.5)	69.0 (44.0 – 87.0)	**0.0011**
% Monocytes	7.0 (3.5- 15.0)	9.0 (3.0 – 20.0)	0.716
Glucose (mmol/L)	3.2 (2.8- 4.4)	3.1 (2.8 – 3.5)	0.072
Protein (g/L)	0.8 (0.6 – 1.2)	0.7 (0.5 – 0.95)	**0.008**

Appendix 3: Univariate Sensitivity Analysis Comparing Confirmed HSV to Confirmed EnV in Immunocompetent Patients (N =42)

	Total (%)		
	**HSV (N = 23)**	**EnV (N= 19)**	**p-value**
CSF analysis (Median, IQR*)			
RBC (x 10^6 /L)	7.0 (1.0 – 16.0)	9.0 (3.0 – 14.0)	0.676
WBC(x 10^6 /L)	346.0 (150.0 – 640.0)	150.0 (42.0 – 365.0)	0.106
% Neutrophils	3.0 (0.0 – 7.0)	20.0 (3.0 – 70.0)	**0.003**
% Lymphocytes	87.0 (71.0 – 94.0)	55.0 (30.0 – 73.0)	**0.0007**
% Monocytes	7.0 (4.0 – 19.0)	11.5 (7.0- 20.0)	0.264
Glucose (mmol/L)	3.2 (2.7 – 5.4)	3.3 (2.6 –3.3)	0.238
Protein (g/L)	0.8 (0.6 – 1.2)	0.4 (0.3 – 0.7)	**0.0008**

Appendix 4A: ROC Curve for Cerebrospinal Fluid Neutrophils for HSV Meningitis/Meningoencephalitis

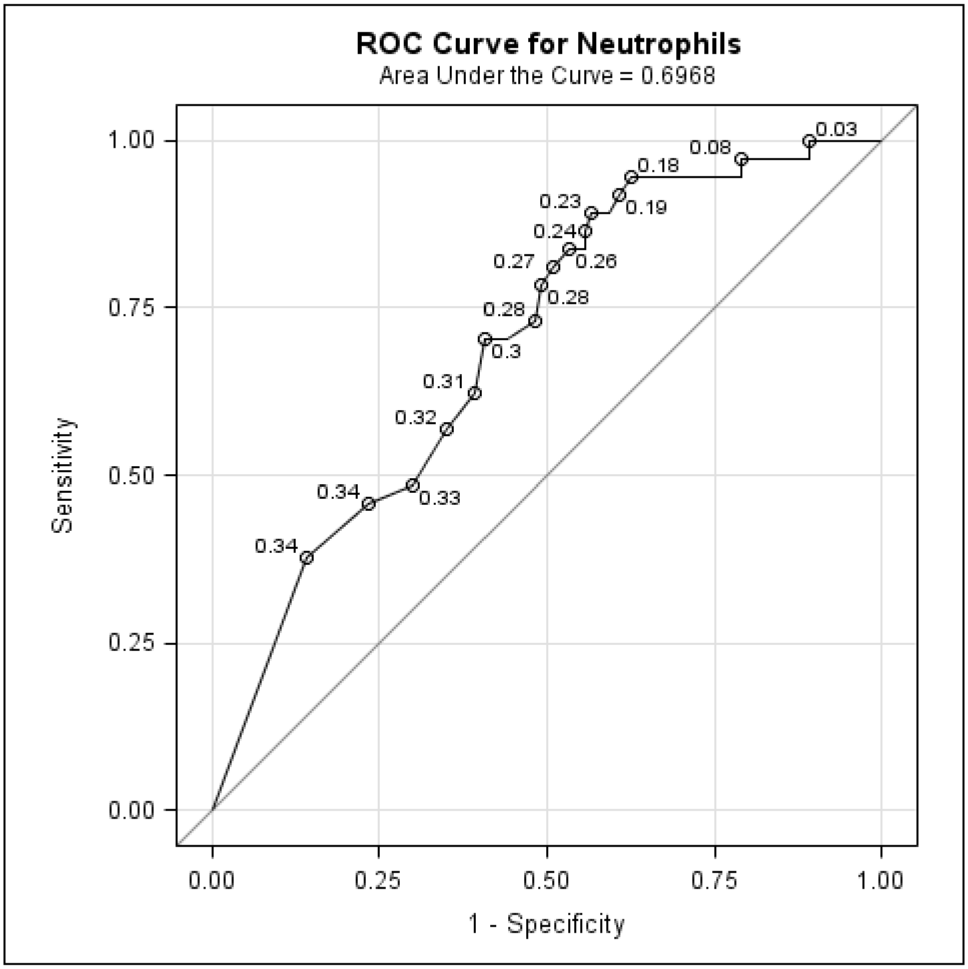

Appendix 4B: ROC Curve for Cerebrospinal Fluid Lymphocytes for HSV Meningitis/Meningoencephalitis

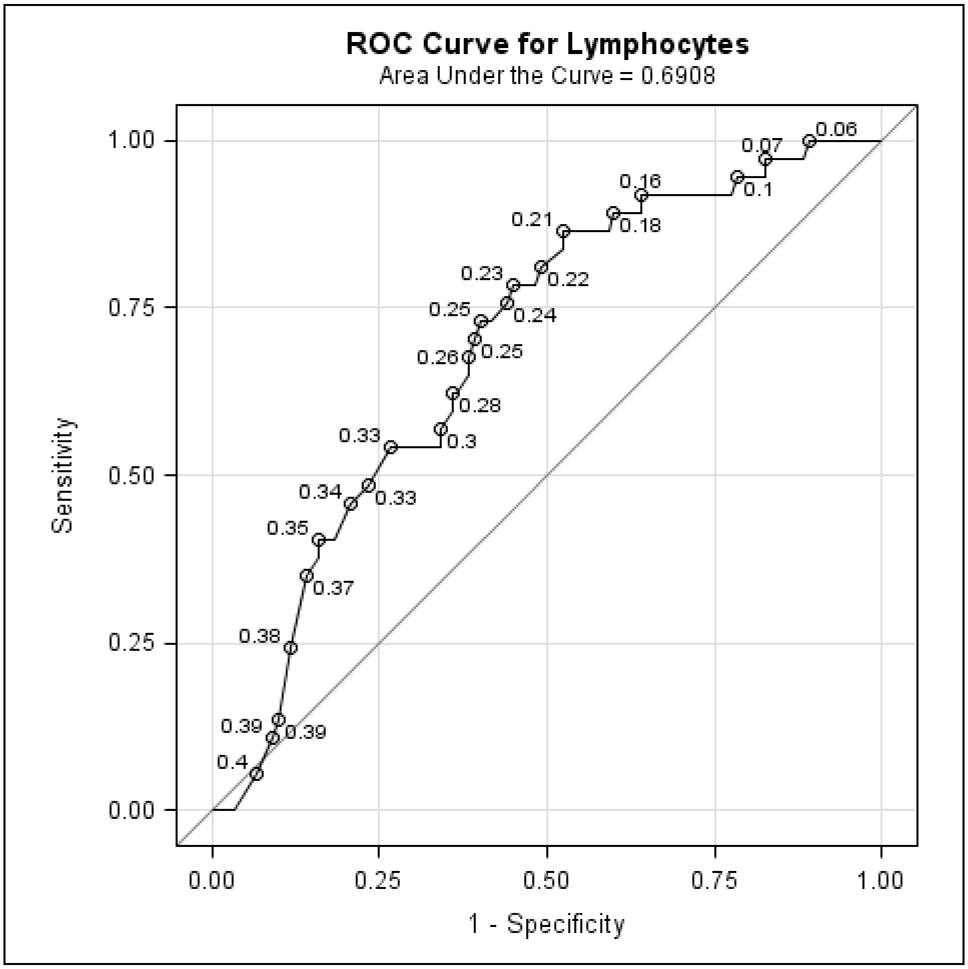

Appendix 4C: ROC Curve for Cerebrospinal Fluid Protein Levels for HSV Meningitis/Meningoencephalitis

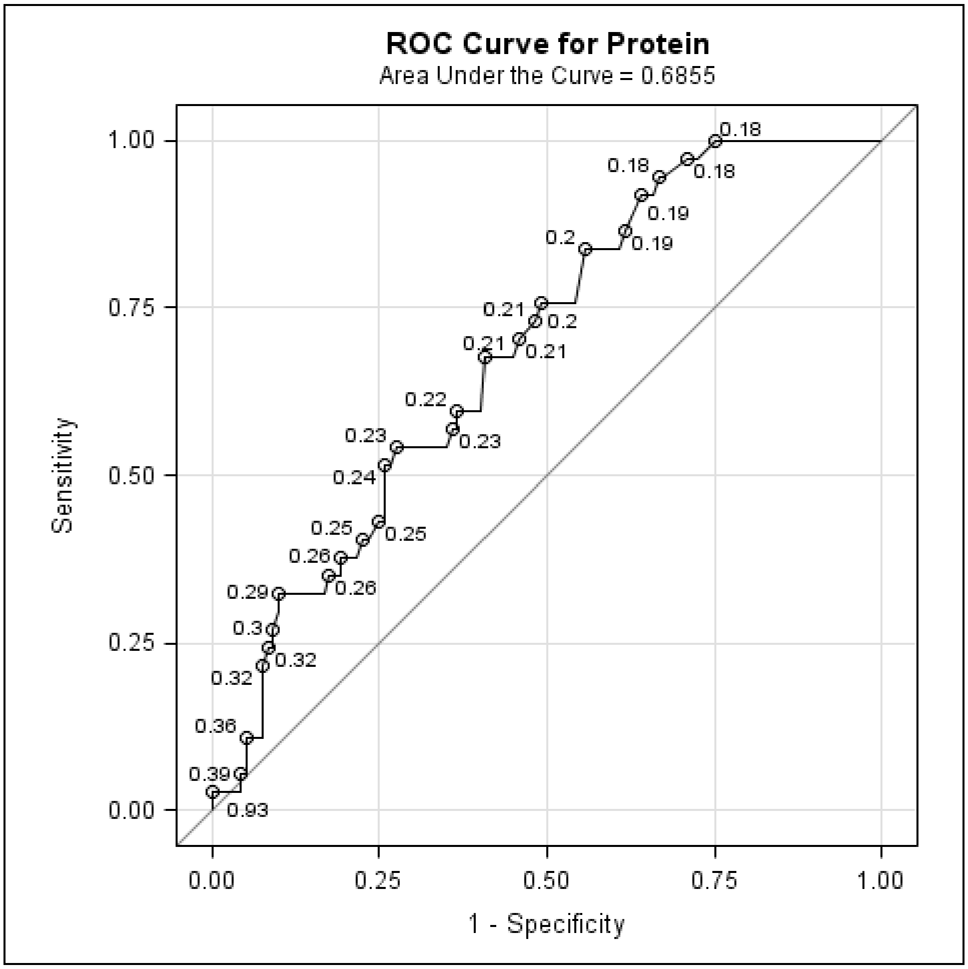

Supplement: Supplementary file 1 — Supplementary Materials for laboratory procedures for polymerase chain reaction testing(Appendix 1); comparison of demographic, clinical characteristics and investigations between patients with herpes simplex virus and enterovirus, excluding pediatric patients (Appendix 2); univariate sensitivity analysis of cerebrospinal fluid of confirmed herpes simplex virus patients to confirmed enterovirus patients in immunocompetent patients (Appendix 3); and receiver operating characteristic curves for cerebrospinal fluid neutrophils, lymphocytes and protein in HSV meningitis/meningoencephalitis (Appendix 4). [file 3463909.f1.pdf]
